# Pleiotropic Effects of a KCNQ1 Variant on Lipid Profiles and Type 2 Diabetes: A Family-Based Study in China

**DOI:** 10.1155/2020/8278574

**Published:** 2020-01-11

**Authors:** Xiaowen Wang, Junhui Wu, Yao Wu, Mengying Wang, Zijing Wang, Tao Wu, Dafang Chen, Xun Tang, Xueying Qin, Yiqun Wu, Yonghua Hu

**Affiliations:** ^1^Department of Epidemiology and Biostatistics, School of Public Health, Peking University Health Science Centre, Beijing, China; ^2^Medical Informatics Center, Peking University Health Science Center, Beijing 100191, China

## Abstract

**Objective:**

The genetic variant rs2237895, located in the Potassium Voltage-Gated Channel Subfamily Q Member 1 (KCNQ1) gene, has been replicated to be associated with type 2 diabetes mellitus (T2DM) susceptibility, but the relationship with lipids is conflicting. Furthermore, the common genetic predisposition to T2DM and lipids was not fully detected.

**Methods:**

In total, 5839 individuals (2220 were T2DM patients) across 2885 families were included. The effect of rs2237895 on T2DM and lipids was estimated using linear regression and logistic regression models after adjustment for multiple covariates. Mediation analysis was then used to test whether KCNQ1 participated in T2DM pathogenesis via lipid-mediated pathways.

**Results:**

Per allele-C of rs2237895 was associated with 17% (11-23%, *P* < 0.001) increased T2DM risk. Moreover, it was correlated with 5% (1-9%, *P* = 0.019), 4% (1-7%, *P* = 0.019), 2% (0-3%, *P* = 0.045), and 2% (0-3%, *P* = 0.009) higher total cholesterol (TC), low-density lipoprotein cholesterol (LDL-C), apolipoprotein A, and apolipoprotein B (Apo-B) concentrations, respectively. Nevertheless, the genetic susceptibility for higher T2DM risk was correlated with higher high-density lipoprotein cholesterol (HDL-C) level (2%, 0-3%, *P* = 0.026). Mediation analysis showed only TC, LDL-C, and Apo-B had small significant mediated effects, with 2.9%, 2.3%, and 3.1% of the total effects of rs2237895 on T2DM being mediated by them, respectively.

**Conclusion:**

KCNQ1 had pleiotropic effects on lipids and T2DM, and the unexpected genetic effect on association of HDL-C with T2DM was observed, indicating the different pathways to lipids and T2DM. Further research studies are needed to verify potential biological mechanisms.

## 1. Introduction

Type 2 diabetes mellitus (T2DM) continues to be an epidemic global health issue across the world. An estimated 425 million adults worldwide had diabetes in 2017, and that figure is expected to reach 642 million by 2040 [1]. T2DM leads to serious complications, such as fatal cardiovascular diseases, diabetic retinopathy, kidney diseases, diabetic neuropathy, microvascular problems, and death, which causes profound distress to patients and exerts a huge burden on healthcare systems [2, 3].

Genetic factors play an important role in the pathogenesis of T2DM [4, 5]. So far, large-scale genome-wide surveys, including genome-wide association studies (GWAS) and whole genome and exome sequencing, have identified more than 200 loci robustly associated with T2DM risk [6, 7]. KCNQ1 (Potassium Voltage-Gated Channel Subfamily Q Member 1), encoding the pore-forming voltage-gated K^+^ channel subunit KvLQT1, is involved in the repolarization of the cardiac action potential as well as water and salt homeostasis [8] and insulin secretion [9]. Rs2237895, a nonsynonymous single nucleotide polymorphism (SNP) in KCNQ1, has been replicated to be associated with T2DM susceptibility and insulin secretion impairment in groups of different ancestries, including East Asian populations, European populations, and American Indians [10–12].

Previous studies have explored the relationships between KCNQ1 SNPs and lipid profiles, including triglycerides (TG), total cholesterol (TC), low-density lipoprotein cholesterol (LDL-C), and high-density lipoprotein cholesterol (HDL-C), but with conflicting results. It has been demonstrated that KCNQ1 gene was associated with lipid parameters, including TG, HDL-C, and apolipoprotein A (Apo-A) among middle-aged Chinese Han population [13]. Another study in general Chinese populations demonstrated a variant in KCNQ1 was associated with TG level but no associations for TC and LDL-C levels [14]. Similar to this, data from a Mexican population suggested significant associations between KCNQ1 and TG and HDL-C [15]. However, no associations were detected in a Mongolian population between two genetic variants on KCNQ1 and any other lipid traits (TC, HDL-C, LDL-C, and TG) [16]. A study conducted in the Turkish Cypriot population also showed no associations between the genotypes of six KCNQ1 polymorphisms and the lipid metabolism [17].

Lipid metabolism is tightly intertwined with glucose metabolism, insulin secretion, and T2DM [2, 18, 19]. Dyslipidemia is a modifiable and independent risk factor for T2DM [2], and there is impairment in lipid metabolism in T2DM patients [19]. However, the genetic predisposition to both lipid profiles and T2DM was not fully detected. Several lines of evidence have been gradually exploring the common genetic signatures [20–25]. Results from the Population Architecture using Genomics and Epidemiology (PAGE) Study in African Americans identified several pleiotropic variants, including APOC1 rs12721054 and LPL rs10096633 in association with glucose, TG, and HDL-C levels [25]. It was observed that PSD3 was associated with obesity, T2DM, and HDL-C level in an American population [20] and PLA2G6 region was associated with T2DM and TG levels in Chinese [21]. The GCKR rs780094 polymorphism was found to be associated with insulin resistance, susceptibility of T2DM, and increased TG levels in Japanese population [23]. A GWAS on Arab individuals from Kuwait identified several genetic risk variants linked to T2DM and lipid profiles [24]. What is more, there were interethnic differences in the biological relationships underlying the lipids–diseases association due to different physiologic and genomic factors [26]. Chinese adults currently experienced a high prevalence of abnormal serum lipid levels, where high TG, TC, and LDL-C rates and low HDL-C rates were presented [27]. The complicated genetic background, pathogenesis, and regulation of lipid metabolism and T2DM were not fully elucidated in Chinese.

Therefore, the aim of the present study was to explore the associations between rs2237895 and lipid profiles (TC, TG, LDL-C, HDL-C, Apo-A, apolipoprotein B (Apo-B)) and T2DM in Chinese to interpret the pleiotropic genetic effects of KCNQ1.

## 2. Materials and Methods

### 2.1. Study Population

The study population was part of the Fangshan/Family-based Ischemic Stroke Study in China (FISSIC) program from 2005 to 2017 in Fangshan district, in the southwest of downtown Beijing, China. The relevant details of the FISSIC program have been described before [28]. Until the end of 2017, 8323 individuals in 5727 families were recruited using the proband-initiated contact method. In the present study, cases were eligible for inclusion if they met all of the criteria as follows: (i) diagnosed as T2DM ≥ 40 years old; (ii) without a history of type 1 diabetes mellitus; (iii) survey data and biological data (including biochemical indicators and blood samples) were available; and (iv) without serious diabetic comorbidities and major mental illnesses. Controls were included if they met the following criteria: (i) age ≥ 40 years at the survey time and (ii) without a confirmed diagnosis of T2DM at the survey time. Finally, a total of 5839 participants (2220 T2DM patients and 3619 controls) were included. See [Supplementary-material supplementary-material-1] in the supplementary material for the study population selection. Each participant received written informed consent. This study has been approved by the Ethics Committee of Peking University Health Science Center (IRB00001052-13027), Beijing, China (22 July 2013).

### 2.2. Assessment of Phenotypes and Covariates

Fasting for at least 8 hours, fasting venous blood was drawn for biochemical collection and measurements. Six common lipid profiles were TC, TG, LDL-C, HDL-C, Apo-A, and Apo-B. The status of T2DM was defined if at least one of the criteria was met: (i) fasting plasma glucose (FPG) ≥ 126 mg/dL, (ii) hemoglobin A1c (HbA1c) > 6.5%, or (iii) ongoing antidiabetic medication therapy. Other information was obtained by structured questionnaires and physical examinations. The questionnaires were completed by face-to-face interviews, including the basic characteristics (age, sex), smoking and drinking status (smokers and nonsmokers; drinkers and nondrinkers), medical history (diabetes, types of diabetes, diabetic complications, hypertension, and coronary heart disease), and medication use history. The height and weight were measured by the physical examinations to calculate BMI (weight/height^2^ (kg/m^2^)).

### 2.3. Genotyping

Amplification of the genomic DNA fragment was performed using an Amplicon ThermoEx 500 PCR instrument followed by a mass spectrometry system using a matrix-supported laser release/ionization time-of-flight mass spectrometry (MALDI-TOF MS) for high-throughput, fast, and accurate genotypic analysis. Negative (blank) and positive controls (standard reference of C : C, A : C, and A : A genotype at rs2237895) were provided. To validate genotyping procedures, the reproducibility of the 5% random sample reached 100%, and the call rate was over 95%.

### 2.4. Statistical Analysis

Continuous variables were described as the mean ± standard deviation, and the paired *t*-test was adopted to compare continuous variables. Categorical variables were described as proportions, and McNemar's chi-squared test was used to compare groups. Hardy-Weinberg equilibrium (HWE) was tested using chi-squared statistic. HWE *P* value for rs2237895 was 0.156, showing no violation for rs2237895. Then, an additive genetic model was assumed for rs2237895 where the genotype value was coded as the count of risk allele-C. The additive model showed a linear relationship between the number of risk alleles and disease risk.

To estimate the association of rs2237895 with the lipid parameters, we applied a general linear regression model. Associations between genotype and T2DM (dichotomous outcomes) were tested using a logistic regression model. All models were adjusted for age, sex, smoking and drinking status, BMI, hypertension, and coronary heart disease. The results were expressed as the percent change (PC) and 95% confidence intervals (CIs) in per allele-C of rs2237895 associated with increase in lipid concentrations and T2DM risk or each 1 mmol/L lipid concentrations associated with increase in T2DM risk. Subgroup analyses were conducted separately in subjects with BMI ≥ 24 and BMI < 24, smokers and nonsmokers, and drinkers and nondrinkers.

Counterfactual-based mediation analysis was then used to test whether KCNQ1 participated in the pathogenesis of T2DM via lipid-mediated pathways. The mediation contains two regressions. The first for the binary outcome (T2DM) was regressed on the exposure (rs2237895), the proposed mediator (lipid parameters), and other relevant covariates. The other regression was for the mediator, regressing on the exposure and the same covariates. Both regressions could obtain the effects mediated by lipid (indirect effect) and by pathways other than those involved in mediators (direct effect). The proportion mediated was equal to OR^d^∗(OR^d^ − 1)/(OR^d^∗OR^i^ − 1), where OR^d^ is the direct effect odds ratio and OR^i^ is the indirect effect odds ratio [29]. All statistical tests were two-tailed, and a *P* value < 0.05 was considered significant. All analyses were performed using R Programming Language (V.3.2.2, R Development Core Team).

## 3. Results

### 3.1. Description of the Study Population

The baseline characteristics of study population were summarized according to the T2DM patients and control groups (Table 1). Individuals who suffered T2DM were more likely to be women and older and to have higher body mass index (BMI) and TG but lower TC, LDL-C, HDL-C, Apo-A, and Apo-B concentrations. The T2DM patients were more susceptible to have hypertension and coronary heart disease, while more controls were drinkers and smokers.

### 3.2. Correlation between Different Lipid Parameters and T2DM

Most lipid parameters, including TC, TG, LDL-C, HDL-C, and Apo-B, were significantly associated with T2DM risk. Each 1 mmol/L increase in TC, TG, LDL-C, and Apo-B conferred a 6% (2-10%, *P* = 0.003), 14% (11-18%, *P* < 0.001), 7% (2-12%, *P* = 0.006), and 27% (9-48%, *P* = 0.003) increased risk of T2DM, respectively. In contrast, each 1 mmol/L increase in HDL-C conferred a 25% (-34-16%, *P* < 0.001) decrease in T2DM risk ([Supplementary-material supplementary-material-1]).

### 3.3. Pleiotropic Effect on Lipid Parameters and T2DM

The relations between rs2237895 and different lipid parameters and T2DM are presented in Table 2. There were significantly positive associations between rs2237895 and TC, LDL-C, HDL-C, Apo-A, and Apo-B after adjustment under an additive model. Per allele-C of rs2237895 was associated with 5% (1-9%, *P* = 0.019), 4% (1-7%, *P* = 0.019), 2% (0-3%, *P* = 0.026), 2% (0-3%, *P* = 0.045), and 2% (0-3%, *P* = 0.009) higher levels of TC, LDL-C, HDL-C, Apo-A, and Apo-B, respectively. Under an additive model, per allele-C was significantly associated with 17% (11-23%, *P* < 0.001) increased T2DM risk after adjustment for the covariates. When adding the lipid parameters as covariates, a positive correlation was still found between rs2237895 and T2DM (18%, 11-24%, *P* < 0.001).

Subgroup analysis were established to explore whether BMI, smoking, and drinking status might influence the pleiotropic effects on lipid and T2DM. It demonstrated that the associations between rs2237895 and some lipid parameters disappeared. However, a positive correlation was still found between rs2237895 and T2DM. Compared with the individuals with BMI < 24, per allele-C of rs2237895 was associated with 18% (4-34%, *P* = 0.011) increased risk of T2DM in BMI ≥ 24. Per allele-C of rs2237895 was associated with 23% (13-34%, *P* < 0.001) increased risk of T2DM in smokers compared with 14% (5-23%, *P* = 0.001) increase in nonsmokers. In drinkers, per allele-C of rs2237895 was associated with 20% (12-29%, *P* < 0.001) increased risk of T2DM, compared to 11% (0-24%, *P* = 0.042) increase in nondrinkers after adjusting for conventional risk factors ([Supplementary-material supplementary-material-1]).

We further conducted the mediation analysis to test whether KCNQ1 directly involved in the pathogenesis of T2DM or through lipid pathways. In the mediation analysis, the models for lipid parameters and T2DM were combined to calculate the indirect effect explained by lipid parameters and the direct effect exerted via other pathways. The results showed evidence for a direct effect, and the indirect effect was negligible. Only TC, LDL-C, and Apo-B showed small significant mediated effects, with 2.9%, 2.3%, and 3.1% of the total effects being mediated by TC, LDL-C, and Apo-B, respectively (Table 3).

## 4. Discussion

In the present study, we identified rs2237895 was significantly associated with lipid profiles, including TC, LDL-C, HDL-C, Apo-A, and Apo-B, as well as T2DM risk, which presented phenotypic effects on lipid profiles and T2DM in Chinese population. Rs2237895 had mainly a direct effect on T2DM risk, not through the lipid metabolic pathway. This also accorded with an earlier observation, which showed that KCNQ1 might directly participate in the pathogenesis of T2DM but not BMI-mediated pathways [30]. The effect of KCNQ1 on T2DM may be mainly through the process of insulin secretion [14], which might simultaneously stimulate glucose and lipid synthesis [31, 32]. These results corroborated the findings of a great deal of the previous work in other genetic variants. A Korean study showed region 12q24.12 had pleiotropic effect on FPG, independent of its effects on the lipid profile [33]. Another Chinese study showed the effect of PLA2G6 on T2DM was independent from its effect on TG levels [21]. Since there are many genomic regions exerting pleiotropic effects on both lipids and T2DM, further studies are needed to illustrate the clustering in terms of variants, genes, pathways, and actionable targets.

The most notable results showed that the gene-based relationship between HDL-C and T2DM-related variant was opposite to the observational relationship, where rs2237895 C allele was associated with higher HDL-C concentration but simultaneously higher T2DM risk. Interestingly, previous study had also reported this paradox that the genetic predisposition to dyslipidemia showed pleiotropic lowering effects on glucose traits or T2DM risk. Two large independent prospective cohorts observed higher TG, TC, and LDL-C risk scores, or lower HDL-C risk scores at the genetic level were correlated with lower glucose-related traits, such as FPG, HbA1c, and homeostasis model assessment of estimated insulin resistance (HOMA-IR) [34]. Women's Genome Health Study (WGHS) demonstrated that a weighted 40-SNP TG genetic risk score (GRS) was inversely associated with incident T2DM [35]. Moreover, some studies also suggested that there might be no association between HDL-C-related genes and T2DM. A case-control study in Netherlands found that though significant HDL-C increases of CETP SNP rs3764261 were observed, there was no effect on any measure of insulin resistance or T2DM incidence [36]. GRS analysis using all 21 established HDL-C variants genotyped in full-heritage Pima Indians identified significant associations with HDL-C but not with HOMA-IR and T2DM, but when using a refined GRS, significant associations were observed. Two Mendelian randomization studies concluded genetically reduced HDL-C did not associate with increased risk of T2DM [37, 38]. Another Mendelian randomization analysis showed higher HDL-C seemed to be protective against increasing FPG but not against T2DM, and different size of HDL subfractions might have independent associations with glucose [39]. Since genetic data relating genes influencing lipid levels with glycemic control and risk of T2DM were conflicting, further studies, especially additional information from high-resolution metabolomics, were warranted to explore the genetic architecture of lipids and T2DM.

In the subgroup analysis, the associations between rs2237895 and some lipid parameters disappeared, which might result from insufficient sample size and inadequate power [40]. However, the relationships between rs2237895 and T2DM were still stable, but individuals with BMI ≥ 24, smokers, and drinkers had increased T2DM risk. Previous studies conducted in Chinese population revealed that KCNQ1 was associated with obesity in Chinese T2DM patients [30, 41]. Meta-analysis of GWAS in East Asian ancestry populations identified BMI-associated loci near the KCNQ1, indicating the genetic basis of obesity was relevant to this gene [42]. What is more, another variant in KCNQ1 polymorphisms (rs151290) has been found to be associated with increased risk of T2DM, especially in smokers and alcohol drinkers [43]. The underlying mechanisms were still unrevealed. Three cytosine-phosphate-guanine (CpG) sites within intron 11 of KCNQ1 had been identified that were differentially methylated in current smokers compared with never smokers. cg26963277 was identified as the driving CpG site associated with tobacco smoking at KCNQ1, and KCNQ1 rs231356 T allele was associated with lower methylation of cg26963277 and an increased risk of T2DM. Smoking may increase the risk of T2DM through decreased methylation at KCNQ1 [44]. Studies conducted in rodents suggested that genes encoding the KCNQ1 (Kv7) channels were connected with alcohol consumption, preference, and acceptance [45, 46]. Altering Kv7 channel function can influence dopamine transmission, and alcohol-preferring strains decreased basal levels of dopamine, leading to acute alcohol challenges and ethanol-seeking behaviors [47]. These findings might be clinically useful in disease prevention that T2DM patients with risk allele were supposed to reduce weight and cease to smoke and drink.

The main strength of the study was from the family-based design, which offered similar environmental exposure, thus attenuated population stratification [48]. Although some people were removed from the recruited participants, the characteristics between the two groups were similar. The first limitation was that the participants in the study were recruited from a local community from Northern China. Cautions should be paid when generalizing to other populations. Second, in the mediation analysis, there might be some unmeasured mediator-outcome confounders, such as diet and physical activity, resulting in misestimation of the direct and indirect effects. But the main conventional risk factors had been considered. There is abundant room for further progress in determining the comprehensive genetic background and underlying molecular pathways of lipid metabolism and T2DM.

## 5. Conclusions

In conclusion, KCNQ1 SNP rs2237895 had pleiotropic effects on lipids (TC, LDL-C, HDL-C, Apo-A, and Apo-B) as well as T2DM risk. It was remarkable that we observed paradoxical genetic association of HDL-C with T2DM, where the genetic susceptibility for higher T2DM risk was correlated with higher HDL-C level. Our findings added important evidence for the genetic predisposition to both lipids and T2DM.

## Figures and Tables

**Table 1:** Baseline characteristics of individuals with type 2 diabetes (T2DM) and control subjects.

Characteristics	T2DM	Controls
Number	2220	3619
Age (years)	59.5 ± 8.7	56.4 ± 11.4
Men (%)	43.8	53.1
BMI (kg/m^2^)	26.5 ± 3.64	26.0 ± 4.80
TC (mmol/L)	3.08 ± 1.27	3.24 ± 1.03
TG (mmol/L)	1.72 ± 1.69	1.50 ± 1.22
LDL-C (mmol/L)	2.19 ± 1.00	2.35 ± 0.85
HDL-C (mmol/L)	0.91 ± 1.68	1.02 ± 0.40
Apo-A (mmol/L)	1.06 ± 0.39	1.16 ± 0.43
Apo-B (mmol/L)	0.75 ± 0.36	0.78 ± 0.25
Drinker (%)	30.0	38.0
Smoker (%)	42.0	48.1
Hypertension (%)	76.4	66.7
Coronary heart disease (%)	28.9	27.5
No. of rs2237895 C alleles		
0	39.5	46.4
1	46.5	42.7
2	14.0	10.9

BMI: body mass index; TC: total cholesterol; TG: triglyceride; LDL-C: low-density lipoprotein cholesterol; HDL-C: high-density lipoprotein cholesterol; Apo-A: apolipoprotein A; Apo-B: apolipoprotein B. Notes: continuous variables were reported as mean ± standard error. Differences between cases and controls were analyzed using the paired *t*-test and McNemar's chi-squared test. All tests showed significant *P* values (*P* < 0.001).

**Table 2:** Association between KCNQ1 rs2237895 and different lipid parameters and T2DM.

Variables	*β* (SE)	PC (%) (95% CI)	*P*
Lipid parameters			
TC	0.049 (0.021)	5.01 (0.80, 9.38)	**0.019**
TG	-0.018 (0.028)	-1.75 (-7.03, 3.82)	0.530
LDL-C	0.039 (0.016)	3.93 (0.64, 7.33)	**0.019**
HDL-C	0.015 (0.007)	1.51 (0.18, 2.86)	**0.026**
Apo-A	0.015 (0.007)	1.51 (0.04, 3.01)	**0.045**
Apo-B	0.015 (0.006)	1.51 (0.38, 2.66)	**0.009**
T2DM			
Model 1	0.154 (0.028)	16.65 (10.43, 23.22)	**<0.001**
Model 2	0.163 (0.028)	17.64 (11.30, 24.35)	**<0.001**

*β*: estimate; SE: standard error; PC: percentage change; CI: confidence interval; TC: total cholesterol; TG: triglyceride; LDL-C: low-density lipoprotein cholesterol; HDL-C: high-density lipoprotein cholesterol; Apo-A: apolipoprotein A; Apo-B: apolipoprotein B; T2DM: type 2 diabetes mellitus. Notes: models for lipid parameters and T2DM model 1 were adjusted for age, sex, smoking and drinking status, hypertension, coronary heart disease, and body mass index. T2DM model 2 was adjusted for age, sex, smoking and drinking status, hypertension, coronary heart disease, body mass index, TC, TG, LDL-C, and HDL-C. *P* values < 0.05 are shown in bold.

**Table 3:** Mediation analysis of the association between the KCNQ1 rs2237895 and T2DM risk mediated by lipid parameters.

Variables	Direct effect OR (95% CI)	Indirect effect OR (95% CI)	Total effect OR (95% CI)	PM (%)
TC	1.04 (1.03, 1.07)	1.01 (1.00, 1.02)	1.05 (1.03, 1.06)	**2.9**
TG	1.05 (1.03, 1.06)	1.00 (0.99, 1.00)	1.05 (1.03, 1.06)	1.3
LDL-C	1.05 (1.03, 1.07)	1.01 (1.00, 1.02)	1.05 (1.03, 1.06)	**2.3**
HDL-C	1.05 (1.03, 1.06)	1.00 (0.99, 1.00)	1.05 (1.03, 1.06)	0
Apo-A	1.05 (1.03, 1.07)	0.99 (0.99, 1.00)	1.05 (1.03, 1.07)	0
Apo-B	1.05 (1.03, 1.07)	1.01 (1.00, 1.02)	1.05 (1.03, 1.07)	**3.1**

OR: odds ratio; CI: confidence interval; PM: proportion mediated; T2DM: type 2 diabetes mellitus; TC: total cholesterol; TG: triglyceride; LDL-C: low-density lipoprotein cholesterol; HDL-C: high-density lipoprotein cholesterol; Apo-A: apolipoprotein A; Apo-B: apolipoprotein B. Notes: all models were adjusted for age, sex, smoking and drinking status, hypertension, coronary heart disease, and body mass index. Lipid parameters with significant indirect effects are shown in bold.

## Data Availability

The data in the Fangshan/Family-based Ischemic Stroke Study in China (FISSIC) program used to support the findings of this study are restricted by the Ethics Committee of Peking University Health Science Center in China in order to protect patient privacy. Data are available from Yonghua Hu (Email: yhhu@bjmu.edu.cn) for researchers who meet the criteria for access to confidential data.

## Supplementary Materials

Supplementary Materials Supplementary Figure 1: the flow chart of the study population selection. Supplementary Table 1: association between different lipid parameters and T2DM. Supplementary Table 2: the associations between KCNQ1 rs2237895 and lipid parameters, T2DM after stratification by BMI, smoking, and drinking habits.
