# Supplementary material for: Pleiotropic Effects of a KCNQ1 Variant on Lipid Profiles and Type 2 Diabetes: A Family-Based Study in China
Source: J Diabetes Res. 2020 Jan 11;2020:8278574. doi: 10.1155/2020/8278574 (PMC6982365; doi:10.1155/2020/8278574)
Supplement: Supplementary Materials — Supplementary Figure 1: the flow chart of the study population selection. Supplementary Table 1: association between different lipid parameters and T2DM. Supplementary Table 2: the associations between KCNQ1 rs2237895 and lipid parameters, T2DM after stratification by BMI, smoking, and drinking habits. [file 8278574.f1.pdf]

Supplementary Files

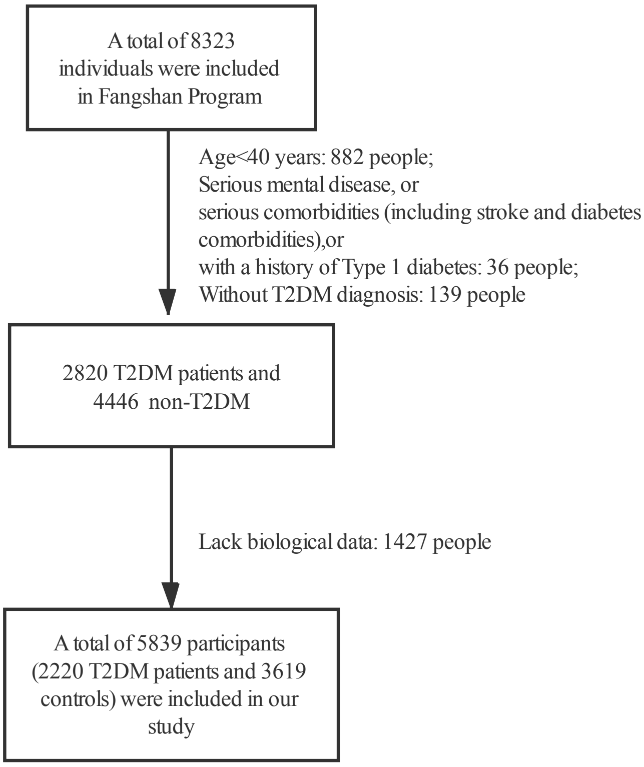

Figure S1. The flow chart of the study population selection.

Table S1: Association between different lipid parameters and T2DM.

Variables	β (SE)	PC, % (95%CI, %)	P
TC	0.056 (0.018)	5.74 (2.11, 9.50)	**0.003**
TG	0.135 (0.016)	14.42 (10.83, 18.13)	**<0.001**
LDL-C	0.065 (0.024)	6.71 (1.89, 11.75)	**0.006**
HDL-C	-0.292 (0.061)	-25.33 (-33.76, -15.83)	**<0.001**
Apo-A	-0.091 (0.055)	-8.73 (-18.04, 1.63)	0.096
Apo-B	0.239 (0.079)	26.93 (8.69, 48.24)	**0.003**

β, estimate; SE, standard error; PC, percentage change; CI, confidence interval; TC, total cholesterol; TG, triglyceride; LDL-C, low density lipoprotein cholesterol; HDL-C, high density lipoprotein cholesterol; Apo-A, apolipoprotein-A; Apo-B, apolipoprotein-B; T2DM, type 2 diabetes mellites.

Notes: All models were adjusted for age, sex, smoking and drinking status, hypertension, coronary heart disease, and body mass index. P values <0.05 were shown in bold.

Table S2: The associations between KCNQ1 rs2237895 and lipid parameters, T2DM after stratification by BMI, smoking, and drinking habits.

Variables	β (SE)	PC, % (95%CI, %)	P		β (SE)	PC, % (95%CI, %)	P
**BMI<24 (n=1667)**				**BMI≥24 (n=4135)**			
**Lipid parameters**							
TC	0.035 (0.025)	3.58 (-1.40, 8.82)	0.162		0.075 (0.044)	7.76 (-1.22, 17.56)	0.093
TG	-0.064 (0.038)	-6.22 (-12.90, 0.96)	0.088		0.067 (0.039)	6.93 (-0.90, 15.37)	0.084
LDL-C	0.032 (0.020)	3.30 (-0.59, 7.34)	0.098		0.047 (0.035)	4.81 (-2.20, 12.32)	0.184
HDL-C	0.020 (0.008)	2.06 (0.55, 3.59)	**0.007**		0.010 (0.016)	0.97 (-2.13, 4.16)	0.544
Apo-A	0.012 (0.009)	1.20 (-0.50, 2.93)	0.169		0.022 (0.017)	2.23 (-1.11, 5.69)	0.193
Apo-B	0.014 (0.007)	1.40 (-0.05, 2.87)	0.059		0.019 (0.010)	1.89 (-0.05, 3.87)	0.057
**T2DM**	0.159 (0.034)	17.20 (9.75,25.15)	**<0.001**		0.165 (0.065)	17.99 (3.93, 33.94)	**0.011**
**Non-smoke (n=3118)**				**Smoke (n=2674)**			
**Lipid parameters**							
TC	0.067 (0.031)	6.98 (0.69, 13.66)	**0.029**		0.045 (0.031)	4.59 (-1.60, 11.17)	0.149
TG	-0.010 (0.041)	-1.03 (-8.70, 7.28)	0.802		-0.032 (0.042)	-3.15 (-10.85, 5.21)	0.449
LDL-C	0.055 (0.023)	5.70 (1.07, 10.55)	**0.015**		0.024 (0.026)	2.41 (-2.75, 7.85)	0.366
HDL-C	0.012 (0.010)	1.17 (-0.71, 3.07)	0.224		0.023 (0.010)	2.35 (0.28, 4.47)	**0.026**
Apo-A	0.022 (0.011)	2.20 (-0.01, 4.47)	0.051		0.016 (0.011)	1.58 (-0.58, 3.79)	0.152
Apo-B	0.020 (0.009)	2.05 (0.25, 3.88)	**0.025**		0.018 (0.010)	1.84 (-0.09, 3.81)	0.062
**T2DM**	0.131 (0.040)	13.94 (5.25, 23.35)	**0.001**		0.208 (0.046)	23.11 (12.52, 34.69)	**<0.001**
**Non-drink (n=3738)**				**Drink (n=2041)**			
**Lipid parameters**							
TC	0.052 (0.036)	5.34 (-1.89, 13.11)	0.152		0.046 (0.028)	4.73 (-0.77, 10.53)	0.094
TG	-0.035 (0.051)	-3.39 (-12.63, 6.82)	0.501		-0.002 (0.036)	-0.20 (-7.07, 7.18)	0.957
LDL-C	0.032 (0.031)	3.28 (-2.76, 9.70)	0.294		0.034 (0.021)	3.41 (-0.73, 7.71)	0.108
HDL-C	0.023 (0.013)	2.30 (-0.23, 4.88)	0.075		0.011 (0.008)	1.15 (-0.50, 2.84)	0.174
Apo-A	0.017 (0.013)	1.76 (-0.72, 4.30)	0.165		0.011 (0.010)	1.07 (-0.88, 3.06)	0.285
Apo-B	0.014 (0.009)	1.37 (-0.35, 3.11)	0.119		0.015 (0.008)	1.46 (-0.13, 3.07)	0.071
**T2DM**	0.108 (0.053)	11.36 (0.39, 23.53)	**0.042**		0.184 (0.037)	20.17 (11.81, 29.14)	**<0.001**

β, estimate; SE, standard error; PC, percentage change; CI, confidence interval; TC, total cholesterol; TG, triglyceride; HDL-C, high density lipoprotein cholesterol; LDL-C,

low density lipoprotein cholesterol; Apo-A, apolipoprotein-A; Apo-B, apolipoprotein-B; T2DM, type 2 diabetes mellites.
Notes: All models were adjusted for age, sex, smoking or drinking status, hypertension, coronary heart disease, and body mass index. P values<0.05 were shown in bold.
